# Supplementary material for: Casein kinase II promotes piRNA production through direct phosphorylation of USTC component TOFU-4
Source: Nat Commun. 2024 Mar 28;15:2727. doi: 10.1038/s41467-024-46882-9 (PMC10978872; doi:10.1038/s41467-024-46882-9)
Supplement: Supplementary file 1 — Supplementary Information [file 41467_2024_46882_MOESM1_ESM.pdf]

Supplementary Information for

Casein kinase II promotes piRNA production through direct phosphorylation of USTC component TOFU-4

Gangming Zhang[1], Chunwei Zheng[1], Yue-he Ding[1] and Craig Mello[1,2]*

[1]RNA Therapeutics Institute, University of Massachusetts Chan Medical School, Worcester, MA 01605, USA

[2] Howard Hughes Medical Institute, Worcester, MA 01605, USA

*Correspondence to: Craig Mello (Craig.Mello@umassmed.edu)

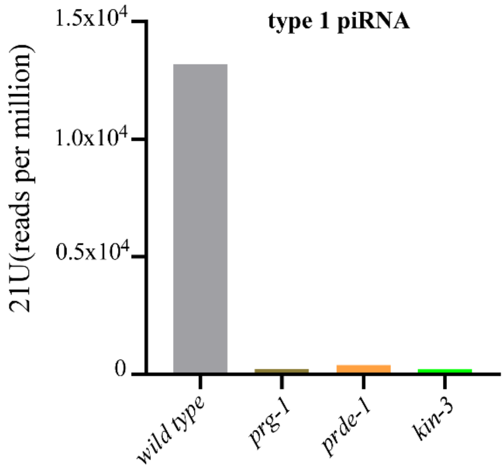

Supplementary Figure 1. CK2 complex affects type I piRNAs level

Bar diagram displaying type 1 21U-RNA abundance in wild type, *prg-1*, *prde-1*, and *kin-3* mutants.

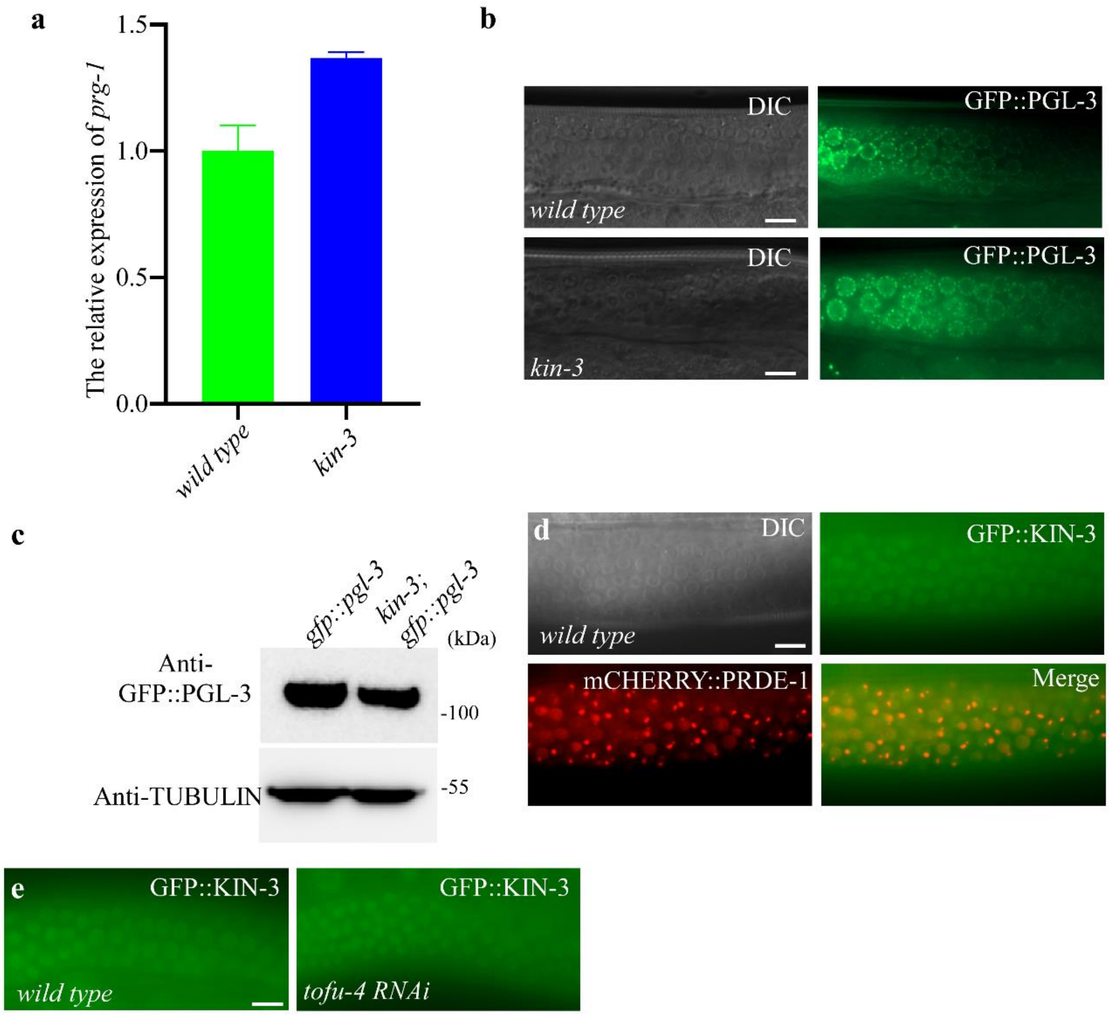

Supplementary Figure 2. CK2 complex does not affect the expression of GFP::PGL-3

a qRT–PCR results of *prg-1* levels in wild type worms and in *kin-3* mutants worms relative to *act-3* mRNA are displayed. mRNA level in wild type worms is set to 1.0. Data are shown as mean ± SD. n = 4 independent biological replicates.

b The expression of GFP::PGL-3 in the wild type worms is similar to that in *kin-3* mutants.

c Protein levels of GFP::PGL-3 are similar in extracts of *kin-3* mutants compared to those of wild type worms. One representative out of 3 independent experiments is shown.

d The colocalization of GFP::KIN-3 and mCHERRY::PRDE-1 in the nucleus is shown.

e The expression of GFP::KIN-3 in wild type and in *tofu-4* RNAi mutants is shown.

Scale bars: 10 μm in **b**, **d** and **e**, for **b**, **d** and **e**, more than 50 worms are counted, the

phenotype is similar. Source data are provided as a Source Data file.

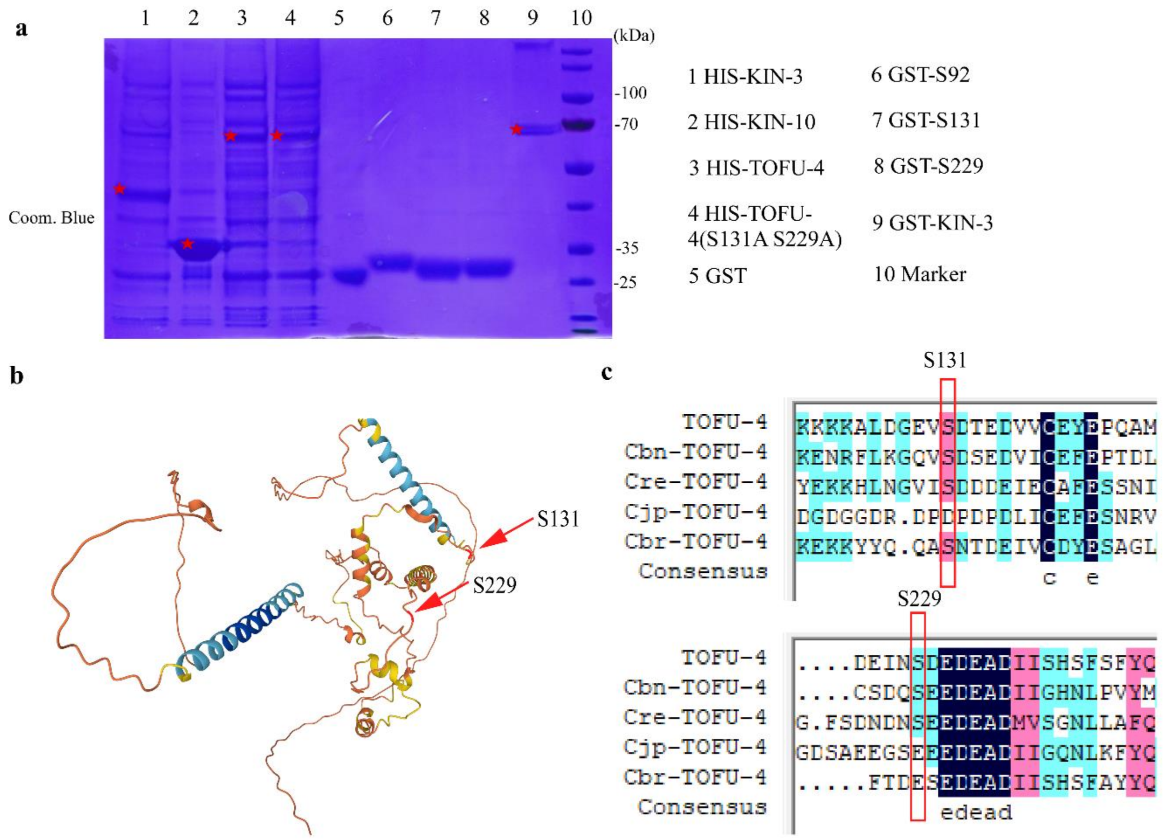

Supplementary Figure 3. TOFU-4 protein structure and sequence homology

a Different proteins purified from *E.coli* are shown. Coom. Blue, Coomassie staining. ★ indicates corresponding fusion proteins.

b Protein structural predictions for TOFU-4 are shown. Protein structure was predicted using the Alphafold (https://alphafold.ebi.ac.uk/). Position of S131 and S229 were indicated by arrowhead.

c Alignment of different nematodes TOFU-4 peptides is shown. Position of S131 and S229 were indicated.

Source data are provided as a Source Data file.

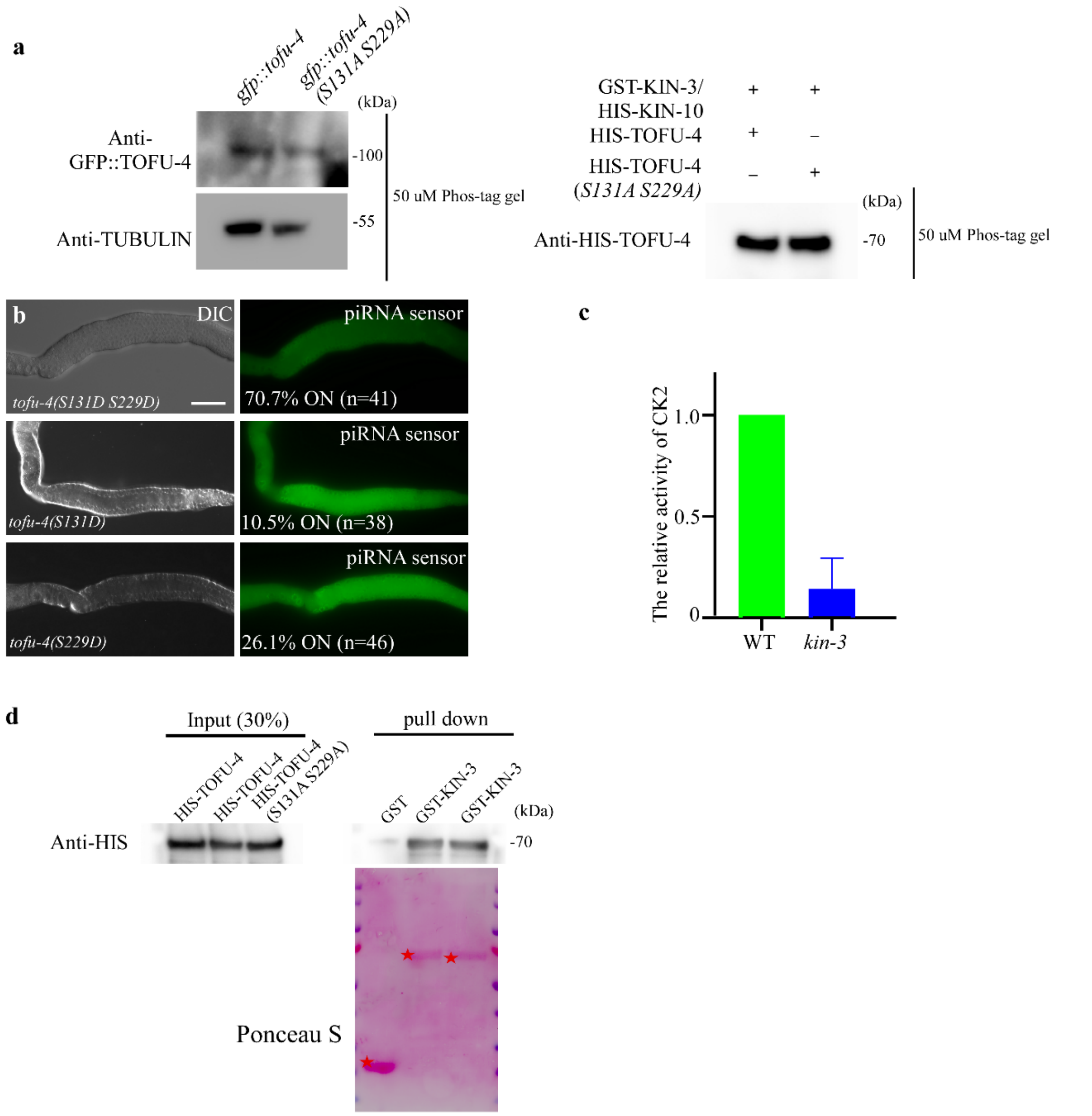

Supplementary Figure 4. CK2 Phosphorylates USTC factor TOFU-4 at S131 and S229

a The results of the Phos-tag gel analyses for *in vivo* TOFU-4 and TOFU-4 (S131A S229A) and *in vitro* HIS-TOFU-4 and phosphorylated HIS-TOFU-4 are presented. One representative out of 3 independent experiments is shown.

b The GFP::CSR-1 transgene is desilenced *tofu-4(S131D S229D)* worms, *tofu-4(S131D)* and *tofu-4(S229D)* worms.

c The activity of CK2 to phosphorylate purified TOFU-4 peptide is largely decreased in *kin-3* mutants. Lysates from wide type and *kin-3* worms of adulthood were used in *in vitro*

phosphorylation assays. The relative activity of CK2 from wide type Day 1worms is set to 1.0. Data is shown as mean ± SD. n = 3 independent biological replicates.

d The GST-KIN-3 interacts with TOFU-4 and TOFU-4(S131A S229A) in a pull-down assay. ★ indicates corresponding fusion proteins. One representative out of 2 independent experiments is shown.

Scale bars: 50 μm for **b** Source data are provided as a Source Data file.
