# Supplementary material for: Casein kinase II promotes piRNA production through direct phosphorylation of USTC component TOFU-4
Source: Nat Commun. 2024 Mar 28;15:2727. doi: 10.1038/s41467-024-46882-9 (PMC10978872; doi:10.1038/s41467-024-46882-9)
Supplement: Supplementary file 3 — Description of Additional Supplementary Information [file 41467_2024_46882_MOESM3_ESM.docx]

**Description of Additional Supplementary Files**

Supplementary Data 1: A list of lethal genes screened.

Supplementary Data 2: Summary of RNAi-based genetic screen of lethal genes using a silenced piRNA sensor.

Supplementary Data 3: List of *C. elegans* strains used in this study.

Supplementary Data 4: List of gRNA and ssOligo donor sequences.

Supplementary Data 5: Reagents used in this paper.
